# An advancement in the synthesis of unique soft magnetic CoCuFeNiZn high entropy alloy thin films

**DOI:** 10.1038/s41598-021-87786-8

**Published:** 2021-04-23

**Authors:** Chokkakula L. P. Pavithra, Reddy Kunda Siri Kiran Janardhana, Kolan Madhav Reddy, Chandrasekhar Murapaka, Joydip Joardar, Bulusu V. Sarada, Rameez R. Tamboli, Yixuan Hu, Yumeng Zhang, Xiaodong Wang, Suhash Ranjan Dey

**Affiliations:** 1 Department of Materials Science and Metallurgical Engineering, Indian Institute of Technology - Hyderabad, Sangareddy, Telangana 502285 India; 2 State Key Laboratory of Metal Matrix Composites, School of Materials Science and Engineering, Shanghai Jiao Tong University, Minhang District, Shanghai, 200240 People’s Republic of China; 3 International Advanced Research Centre for Powder Metallurgy and New Materials (ARCI), Balapur P.O, Hyderabad, Telangana 500005 India

**Keywords:** Materials science, Nanoscale materials, Synthesis and processing

## Abstract

Discovery of advanced soft-magnetic high entropy alloy (HEA) thin films are highly pursued to obtain unidentified functional materials. The figure of merit in current nanocrystalline HEA thin films relies in integration of a simple single-step electrochemical approach with a complex HEA system containing multiple elements with dissimilar crystal structures and large variation of melting points. A new family of Cobalt–Copper–Iron–Nickel–Zinc (Co–Cu–Fe–Ni–Zn) HEA thin films are prepared through pulse electrodeposition in aqueous medium, hosts nanocrystalline features in the range of ~ 5–20 nm having FCC and BCC dual phases. The fabricated Co–Cu–Fe–Ni–Zn HEA thin films exhibited high saturation magnetization value of ~ 82 emu/g, relatively low coercivity value of 19.5 Oe and remanent magnetization of 1.17%. Irrespective of the alloying of diamagnetic Zn and Cu with ferromagnetic Fe, Co, Ni elements, the HEA thin film has resulted in relatively high saturation magnetization which can provide useful insights for its potential unexplored applications.

## Introduction

Nanocrystalline HEA thin films are the new age functional materials for usage in future devices, promoting the technology towards limitless novel applications^1,2^. Unlike the conventional alloys, due to large number of constituent elements in HEAs (the composition of all five elements are equi-atomic or between 5 and 35 atomic%), it leads to exceptional structural properties (high mechanical strength, oxidation and corrosion resistance) and hence, making them promising candidates for functional applications. In this direction, since the last decade, very few surface coating techniques have been adopted such as electrodeposition, sputtering, spray coatings, etc. for fabrication of HEA thin films^2^. Nevertheless, among these surface coating approaches, only few of them are attainable for industrial applications owing to their own limitations. As of late, bulk and thin film HEAs are explored towards magnetic applications to a great extent^3–8^. Yet, recommendations for various functional applications are very limited due to the inferior functional properties compared with conventional soft magnetic materials. Therefore, the complexity remains in synthesizing suitable HEA thin films as soft magnetic materials for functional applications. Though, the existing HEAs majorly show higher saturation magnetization (*M*_*s*_), yet they often do not show lower coercivity (*H*_*c*_) simultaneously^8^. Hence, the current study, focuses on fabricating soft magnetic high entropy alloy thin film using electrodeposition. This new HEA thin films which can potentially fill the void for their magnetic applications such as non-volatile memory, magnetic sensors, nano-transformers, etc. combined with desired excellent structural properties. Very recently FeCoNiCuZn high entropy alloy system has been reported by ball milling^9,10^. In addition, the development of HEAs with a new set of elements having three distinct crystal structures and large variation in their melting points (low melting point element such as Zn), in the form of bulk or thin films have not been explored except ball milling due to the difficulty faced during conventional high temperature alloy making methods. Therefore, fabricating this alloy system in a single step at lower temperatures (close to room temperature) in the form of films or coatings is only possible by electrodeposition in aqueous electrolyte based on their ability to reduce electrochemically which does not involve high temperatures. In addition, optimum utilization of this material can be achieved in the form of thin films where a minimum amount of material is required to engineer the HEAs for prospective applications. Moreover, electrodeposition technique takes few minutes to deposit a single/dual phase HEA. Also, electrodeposition is significantly scalable and economical compared to the existing conventional methods which involve longer durations and high temperatures. Therefore, electrodeposition can be a good substitute fabrication method. Through electrodeposition, several multi-component alloys are produced, yet they are limited only to binary, ternary and quaternary systems^11,12^.

The present study demonstrates new aqueous medium-assisted nanocrystalline dual phase HEA thin films containing unique combination of five elements (Co–Cu–Fe–Ni–Zn) by a cost effective, single step electrochemical deposition which has not been attempted till now. The selection of elements in Co–Cu–Fe–Ni–Zn HEA (Supplementary Fig. S1) were based on their ability to reduce electrochemically in aqueous electrolytes. So far electrodeposition of HEAs by organic and non-aqueous electrolytes, chloride baths resulted barely in crystalline films with single or binary phases. Still, greater part of these HEA thin films were deposited from the same combination of elements which are already reported in bulk HEAs^5,13–15^. There are several challenges such as mixing of five elements, achieving crystallinity, phase, composition, scalability etc. to be considered in a single step deposition. We have eliminated these difficulties in electrodeposition of complex alloys in aqueous medium by applying pulse and engineering other experimental parameters like electrolyte composition, applied potential, pH etc. Application of pulse allows control over composition and microstructure that in turn can tune the properties thereby facilitating large scale production simultaneously.

## Results and discussion

### Designing and fabrication of Co–Cu–Fe–Ni–Zn HEA thin films

Typically, the composition of the alloy predominantly depends on electrolyte composition and processing parameters during electrodeposition. However, in the existing case, electrolyte composition is decided by the anomalous deposition behavior of Fe, Co, Ni elements which are among the principle elements chosen for this multi-component system. Reduction potentials of the five principle elements in the current HEA system follows the order Cu > Ni > Co > Fe > Zn according to their standard reduction potentials. However, at room temperature, deposition of massive Zn with Cu is observed preferentially than Fe, Co and Ni leading to Zn-Cu rich films. This can most likely be due to their easy formation of metal hydrate ions, hydroxide/ligands (ligands form as a result of complexing agents) and their quick dissociation in reduction process in addition to their other multiple reduction sequences at the cathode surface^16,17^. Therefore, composition of the electrolyte has been optimized and depositions were carried out at slightly higher temperature of ~ 45 ºC where Fe, Co and Ni are deposited along with the Cu and Zn, resulting in the formation of multicomponent alloy (MCA) thin films with composition beyond HEA (5–35 atomic %). Hence, the concentration of the electrolyte, complexing agents, buffer have been adjusted (Supplementary Table S1) according to the required composition of the five principle elements (near equi-atomic). This optimization is performed by considering the hydroxide/ligand formation and their dissociation, two step reduction and multiple elements complex formation and their dissociation during the deposition. In the current scenario, complexing agent (sodium citrate) is used to bring the reduction potentials of multiple ions closer and to diminish the formation of metal hydroxides by forming complexes simultaneously. The local pH at the interface was balanced by the addition of boric acid as a buffer. In addition, by taking the anomalous deposition^16,17^ and hydroxide suppression mechanism into consideration, Ni ion concentration is increased compared to Fe and Co. Besides the Ni ion concentration, nickel chloride is also being added in very small amounts to activate the nickel anode and to maintain the Ni ion concentration in the electrolyte throughout the deposition. Further, none of the metal ion concentrations were maintained equally to avoid the multiple ion complexes and their reductions. During the electrodeposition undesired reactions occur such as hydrogen formation due to the partial hydrolysis of metal ions at the cathode, which increases the pH and leads to the precipitation of metal hydroxides.

**Figure 1:**
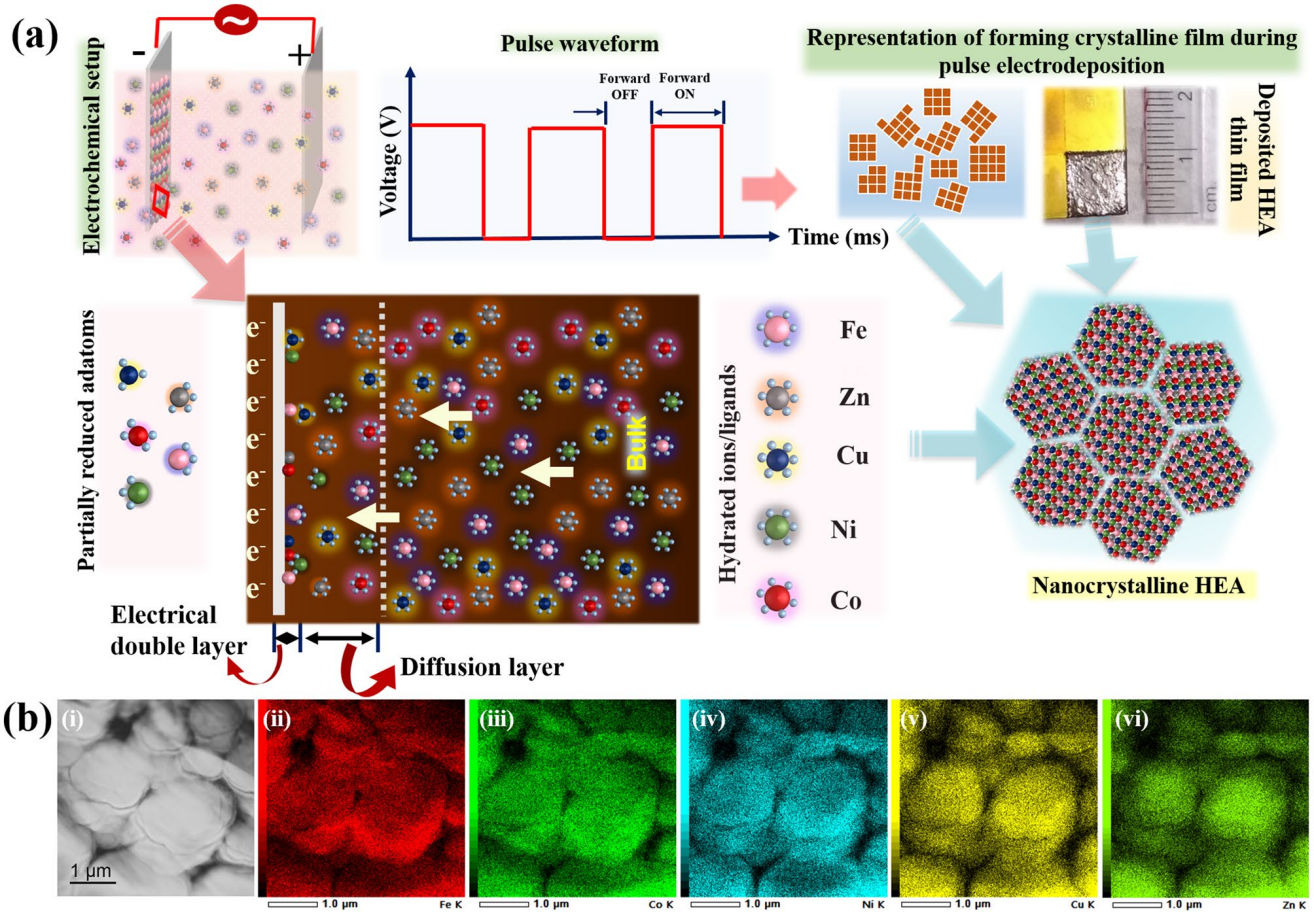
(**a**) Schematic representation of electrochemical setup of reduction of metal ions during pulse electrodeposition of nanocrystalline Co–Cu-Fe–Ni-Zn HEA thin films (**b**) STEM-EDS of HEA thin film showing the elemental mapping of Fe, Co, Ni, Cu and Zn (ii-vi) respectively.

The raise in pH leads to preferential deposition of Zn, as it forms Zn(OH)^+^ and the dissociation of Zn(OH)^+^ is more favourable compared to Fe group elements, whereas Cu has the ability to reduce itself readily. Therefore, the Cu and Zn concentrations are reduced drastically in the electrolyte to limit their activity in this preferential deposition. After Zn, Fe is more active and preferentially covers the surface of the cathode by inhibiting Co and Ni (Fe > Co > Ni). In addition, hydroxide formation is very favourable in Fe, Co and Ni due to the raise in pH. Yet, the dissociation of Fe(OH)^+^ is faster compared to the Co(OH)^+^ and Ni(OH)^+^ which leads to the higher content of Fe > Co > Ni in the deposit. Therefore, co-deposition of five elements becomes more competitive and complex at the cathode-electrolyte interface. However, these simultaneous reactions and reductions/adsorptions of metal hydrate ions, hydroxides/ligands at cathode can be controlled by varying the pulse parameters such as forward ON and OFF timings. During the deposition, anomalous behaviour and/or hydroxide suppression mechanisms are competing factors in the alloy composition and microstructure generation. Yet, the pulse parameters play an important role in the reduction of five metal ions uniformly on the cathode surface (Fig. 1a).

During the forward pulse in pulse electrodeposition technique (potentiostatic mode), all the elements were reduced during the ON time. Further, pH raise at cathode surface due to the presence of OH^−^ ions was minimized because of the presence of buffer and application of OFF time. This maintains the pH in the required range which reduced the preferential metal hydroxide/hydrate ion population at the cathode surface for the next forward pulse ON time. Moreover, due to the OFF time, decline of the diffusion layer thickness results in decreased concentration profile of metal hydroxide/ligands drastically at the cathode surface. Unlike in the conventional direct current deposition, this acutely small diffusion layer accelerates the ions to reach effortlessly from the bulk towards the cathode surface as well. Nevertheless, during the next forward ON time, though all the metal ions reaches the cathode surface, reduction occurs according to their respective sequence based on their dissociation constants. So, throughout the process of pulse ON and OFF times, the preferential surface coverage of metal hydrate can be depleted to a great extent. In addition, pulse ON time followed by OFF time provides the passage of all five metal ions and their reductions at cathode while maintaining the pH at the cathode by supressing the hydrogen entrapment, impurities etc., rapidly at the surface thereby producing uniform composition. In this study the optimized duty cycle > 70% resulting in high quality metallic films with uniform composition. In addition to the composition, application of pulse with an interruption after every forward pulse enables the deposition of all five elements with essential composition having minimal residual stresses especially during the deposition of alloys like HEAs containing multiple elements.

Observations were made using Field emission scanning electron microscope (FESEM) equipped with Energy dispersive X-ray spectroscopy (EDS) where, pulse electrodeposited HEA thin films were found containing fine microstructure with uniform distribution of elements throughout the film (Supplementary Fig. S2). The EDS has been collected from nine locations of the sample and average composition is given (Supplementary Table S2). Composition of HEA thin films were also confirmed at very small length scales in Scanning transmission electron microscopy (STEM) equipped with EDS (Fig. 1b) and the composition observed from STEM-EDS is given in Table 1. From STEM-EDS (Table 1) and FESEM-EDS (Supplementary Table S2), it is observed that the composition of HEA thin films is varied. This difference is probably due to observations made at different length scales and also due to the local heterogeneties which may arise from multiple elements in a concentrated solid-solution, and their packing and atomic-level arrangements^18^. Additionally, as the data obtained from STEM-EDS is from only one location, the data from FESEM-EDS is more reliable as the data has been collected from various locations (approx. 9 locations at 1000x). However, the distribution of Co–Cu–Fe–Ni–Zn is measured to be very uniform and found to be present in the required HEA range thus, the deposited thin films are confirmed to be HEA films.

**Table 1:** STEM-EDS of HEA thin film prepared by pulse electrodeposition (data corresponds to Fig. 1b).

Element	KeV	Weight%	Atomic%
Fe K	6.39	17.84	19.41
Co K	6.92	19.21	19.79
Ni K	7.47	16.59	17.17
Cu K	8.04	21.72	20.76
Zn K	8.63	24.64	22.87
Total		100	100

### Microstructure and phase constituents of Co–Cu–Fe–Ni–Zn HEA thin films

**Figure 2:**
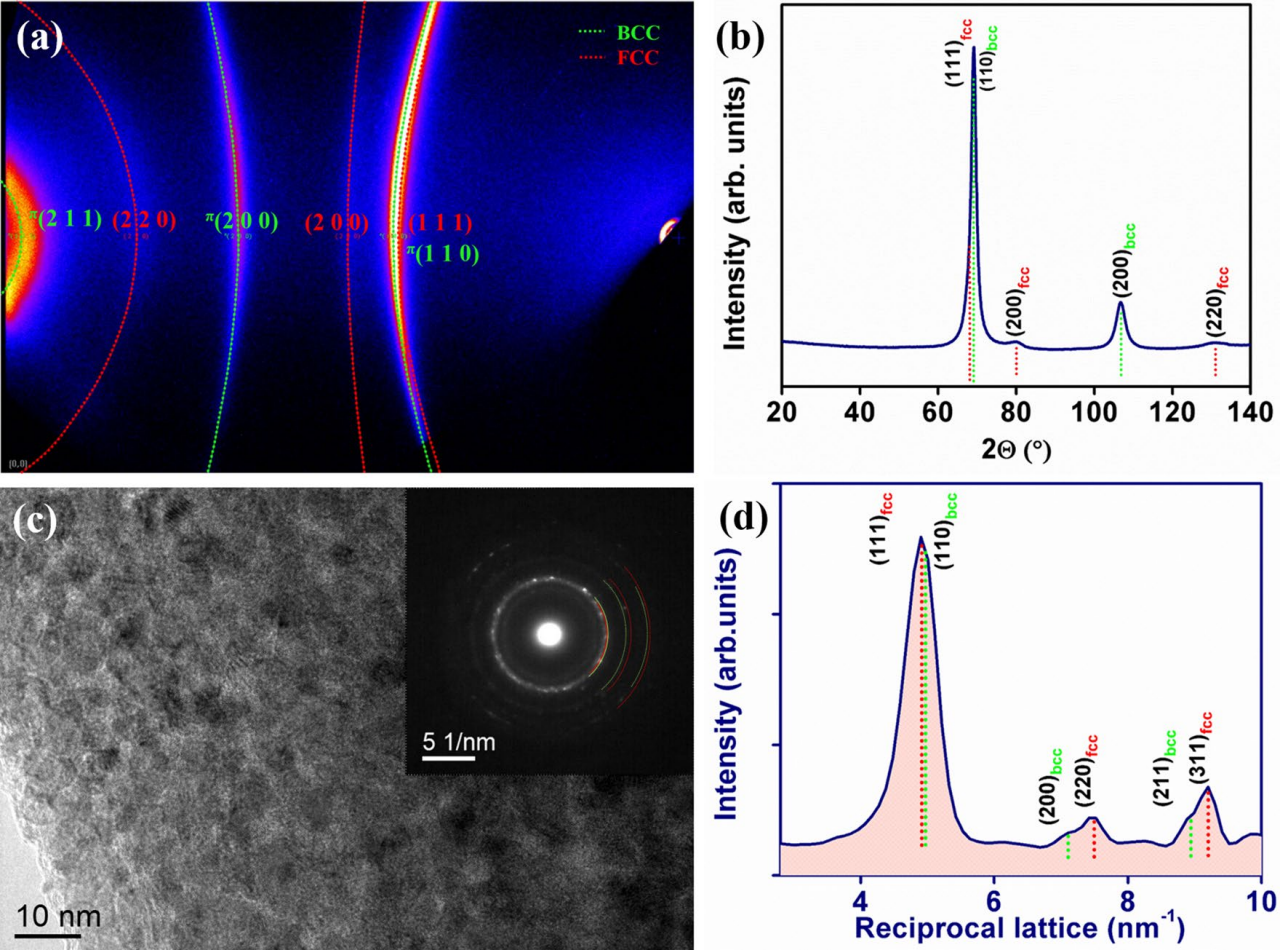
Crystallinity and phase (**a**) 2D X-ray diffraction image and (**b**) X-ray diffraction pattern integrated from 2D X-ray diffractogram confirms the dual phase HEA (**c**) transmission electron micrograph of HEA thin film confirms the nanocrystalline features and (**d**) SAED pattern in reciprocal space confirms the presence of FCC and BCC phases in Co–Cu–Fe–Ni–Zn HEA.

The thin films were characterized for their microstructural features, crystallinity and phase fraction by high-intensity 2D X-ray diffraction system equipped with a Cr source and high resolution transmission electron microscopy (HRTEM). Figure 2a**.** indicates the 2D X-ray diffraction and Fig. 2b. represents the integrated diffractogram from 2D X-ray pattern of HEA thin film indicating the dual face centered cubic (FCC) and body centered cubic (BCC) phases with lattice constants of 3.55 Å and 2.85 Å, respectively. Transmission electron micrograph (Fig. 2c) of HEA thin film confirms nanocrystalline features of the alloy where, the grain size is ranging from ~ 5 to 20 nm and a selected area electron diffraction (SAED) pattern (Fig. 2c-inset) also confirms the dual phase.

**Figure 3:**
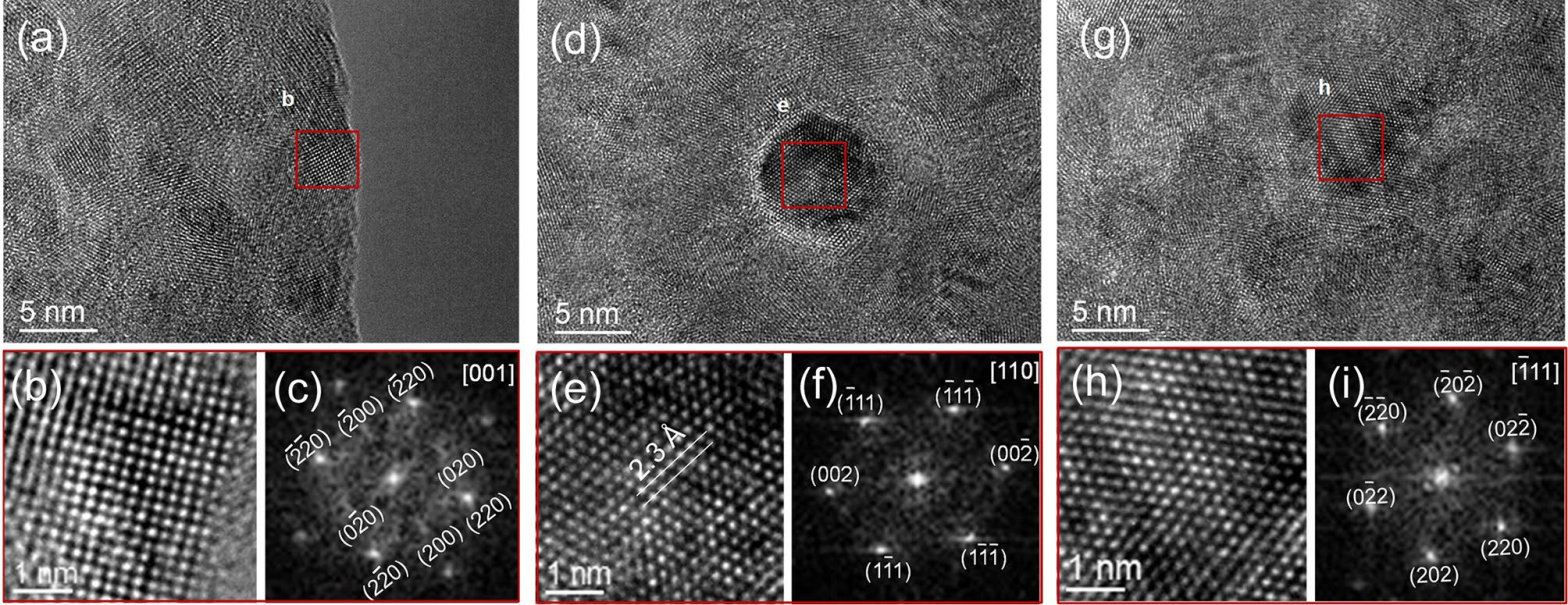
High resolution TEM images of Co–Cu–Fe–Ni–Zn HEA thin films nanocrystalline structure projected for the three principal zone axes of an FCC/BCC phase. (**a**, **d** and **g**) BF-TEM on-axis image of a fine grain size is shown in red boxes. (**b**, **e** and **h**) Illustration of lattice image contains easily interpreted d spacing information. (**c**, **f** and **i**) FFTs taken from image (**c**, **f** and **i**) indexed for BCC/FCC crystals in the beam directions of [001], [110] and [$\overline{1}$11]. Ratios of inter-spacing and the angles are well matched with standard BCC (**c** and **i**) and FCC (**c**, **f** and **i**) diffraction patterns.

In addition, from the 2D X-ray diffraction and reciprocal map (Fig. 2d) indicating the presence of reflections from (111), (200) and (311) planes of FCC and (110), (200) and (221) planes of BCC phases in the thin film which confirms the dual phase. HRTEM images revealed fine nanocrystals (Fig. 3a,d,g) with a grain size of ~ 8 nm. High magnification HRTEM images on-axis (Fig. 3b,e,h) illustrates an atomic lattice structure similar to a cubic crystal with a d-spacing of d_111_ = 2.3 Å (Fig. 3e). Fast Fourier transform (FFT) patterns taken from same regions of images for the major principal zone axis (Fig. 3c, f, i) confirms the FCC and BCC HEA phases. Further high resolution STEM-EDS maps of Fe, Co, Ni, Cu and Zn (Supplementary Fig. S3) confirmed that these nanometer sized grains do not have any different chemical compositions irrespective of FCC and BCC HEA phases. In this study, current HEA has elements with dissimilar crystal structures of BCC, FCC and HCP. However, application of pulse with a precise control over deposition parameters, dual phase HEA thins films were achieved successfully in a single step. FCC/BCC dual phase of these HEA thin films is a trade-off between strength and ductility which is essential in designing the new HEAs.

**Figure 4:**
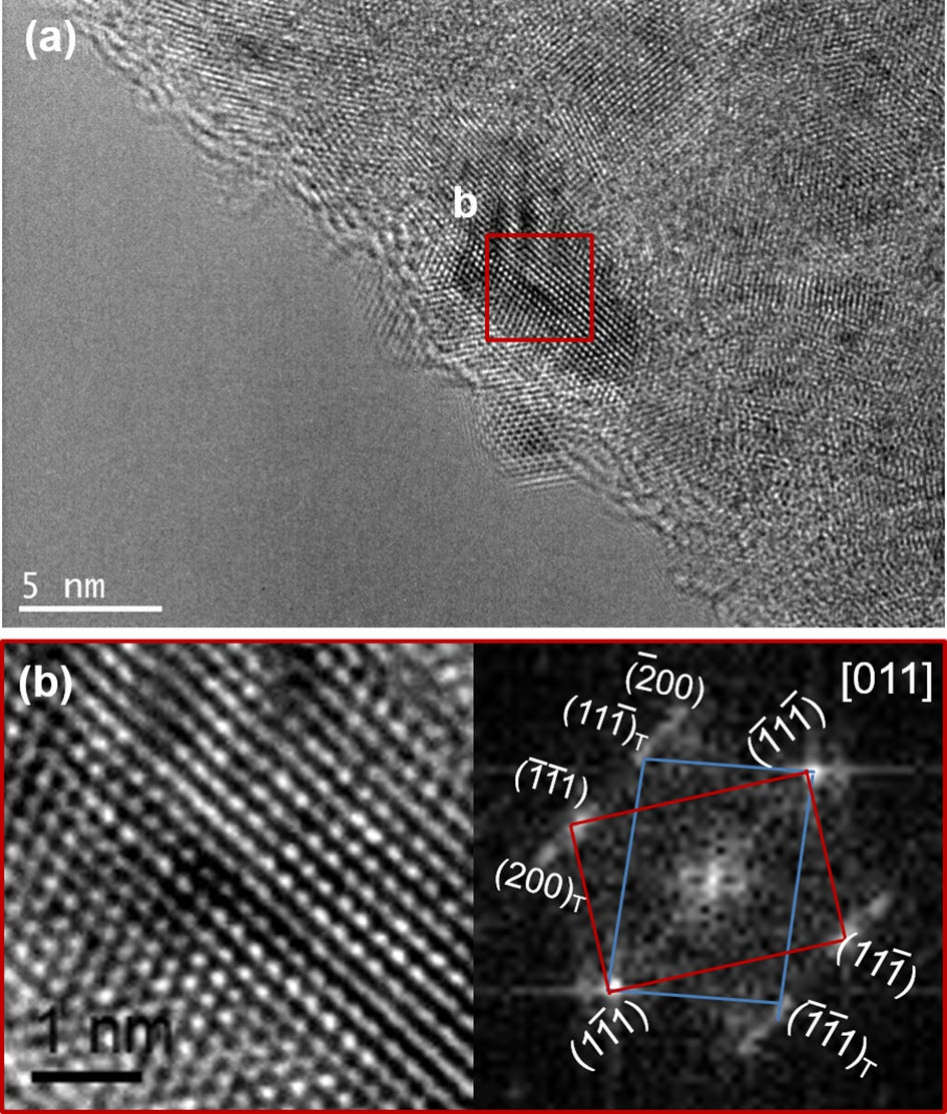
High resolution TEM micrograph of Co–Cu–Fe–Ni–Zn HEA thin film indicating the presence of growth-twins.

Pulse electrodeposition enables more nucleation sites due to the application of regular pulses which leads to nanocrystalline features i.e. finer grain size with globular structure (Fig. 1b). Moreover, multi-component system has various elements with various atomic sizes and crystal structures that generate large amount of stresses and lattice distortions. However, pulses given at regular intervals reduce the residual stresses leading to the nanocrystalline features with good phase stability. In pulsed electrodeposition, the OFF time leads to the relaxation and rearrangement of adsorbed ions, resulting in crystallinity. Yet, deposition of multiple elements having differences in crystal structures and atomic size generates large amount of internal stresses in the deposit which probably may have resulted in lacking the order of deposited elements, leading to the mixed structure of FCC and BCC. In addition, pulse electrodeposition can drive to the formation of twins in the deposit due to the stress–strain relaxation that occurs during the OFF time in deposition^19,20^. Twin formation in the HEA films (Fig. 4) also confirms that the deposited HEA films are having low stacking fault energy (SFE).

### Magnetic behavior

**Figure 5:**
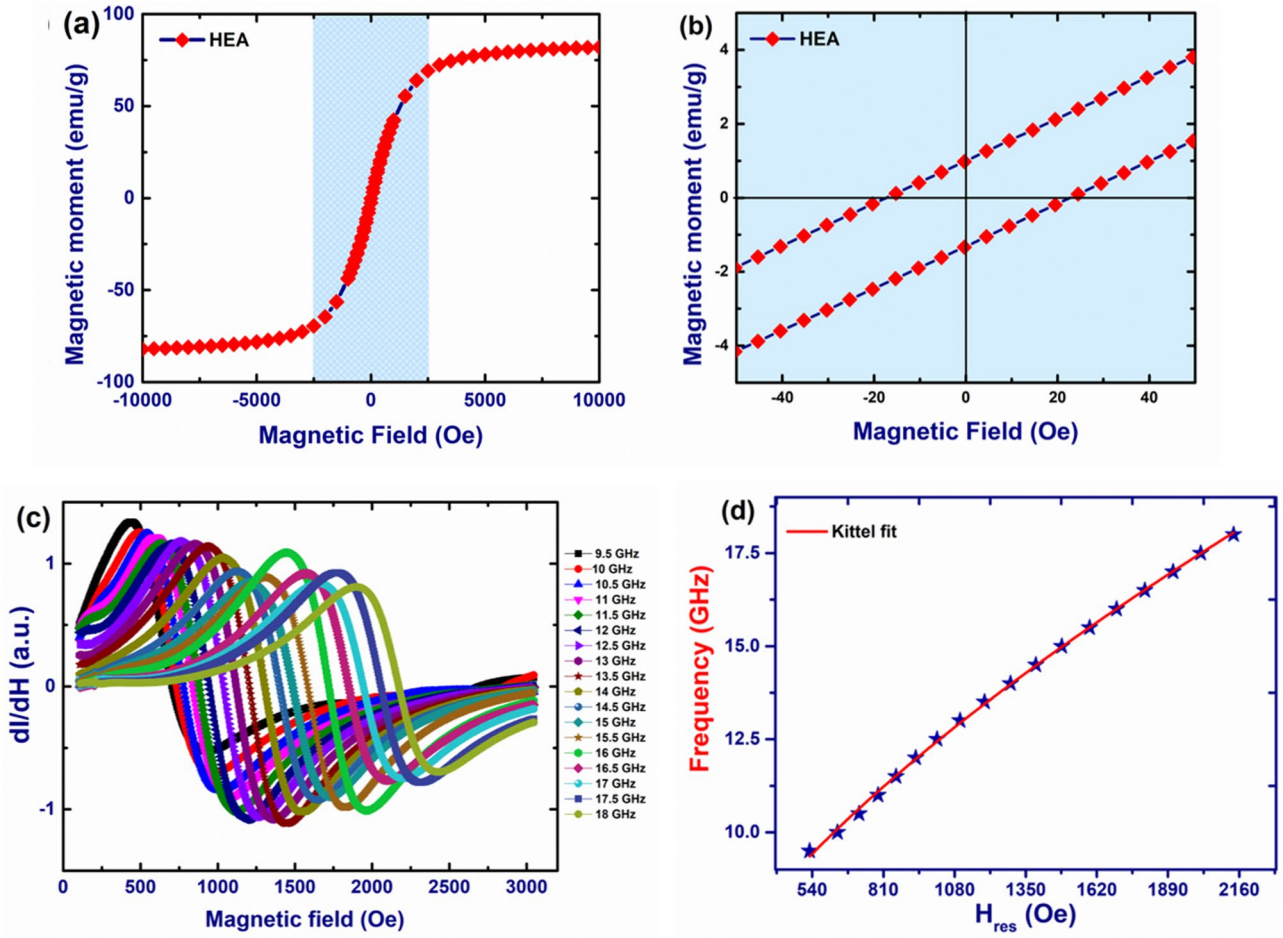
Magnetic properties of HEA thin films (**a**) the hysteresis loop (MH curve obtained from VSM at room temperature) (**b**) magnified hysteresis (**c**) FMR response of HEA thin films at various frequencies (d) Kittel fit.

The magnetic moment of HEA (Fig. 5a) as a function of external magnetic field has been measured by vibrating sample magnetometer (VSM) and are found to exhibit soft magnetic behaviour. The obtained saturation magnetization (*M*_*s*_) of electrodeposited HEA thin film is 82 emu/g (Fig. 5a). Whereas the coercivity (*H*_*c*_) and remanent magnetization (*M*_*r*_) (Fig. 5b) for these films were found to be 19.5 *Oe* and 1.17% respectively, revealing superior magnetic properties when compared to existing reports on the same system^10^. The soft magnetic behaviour with relatively low *H*_*c*_ and low *M*_*r*_ of HEA thin films can be attributed to the crystalline nature of the films.

In addition to the static characterization to obtain hysteresis loops from VSM, magnetization dynamic measurements have also been performed to investigate the microwave properties of the electrodeposited alloy. Ferromagnetic resonance (FMR) technique was used to study the microwave absorption in Co–Cu–Fe–Ni–Zn HEA thin film from 9 to 18 GHz range. For the FMR measurements, a radio frequency magnetic field is injected using a co-planar wave guide into the thin film while a dc magnetic field is applied to induce magnetization precession within the thin film. When a frequency of the magnetization precession matches with external RF field, resonance occurs resulting in microwave absorption.

The resonance frequency (*f*) (Fig. 5c,d and Supplementary Fig. S5) is plotted as a function of resonance field (*H*_*res*_). The ferromagnetic resonance in the current HEA thin films are found to follow Kittel equation (Eq. 1)

1 $$f = \frac{\gamma }{2\pi }\sqrt {\left( {H_{res} + H_{k} } \right)\left( {H_{res} + H_{k} + 4\pi M_{s} } \right)}$$

Here γ/2π is the gyromagnetic ratio, H_K_ is anisotropy field and *M*_*s*_ is the saturation magnetization. As shown in Fig. 5c, the FMR response of HEA thin film clearly shows microwave absorption at various frequencies. By fitting the data obtained from FMR measurements to the Kittel equation, the *4πM*_*s*_ value of the HEA thin film is found to be ~ 16,136 *Oe*. However, in FMR, the results are corresponding to the local region in which the co-planar waveguide is in contact. Therefore, the *M*_*s*_ value extracted from VSM measurements is more appropriate compared to the FMR measurements as VSM takes the whole sample into consideration. Nevertheless, the dynamic measurement results from FMR indicate that the current electrodeposited HEA can have potential applications in microwave devices.

This enhancement in the saturation magnetization is attributed to the addition of Co which has high magnetic moment. Relatively higher *M*_*s*_ in this HEA can be associated with two different factors: (1) composition of the elements and (2) crystalline nature. From the observations made from EDS, HEA thin film is found to be composed majorly of ferromagnetic elements Fe, Co and Ni, compared to Zn and Cu. The higher composition of Fe, Co and Ni magnetic elements in this thin film could be responsible for relatively large *M*_*s*_ even with the addition of diamagnetic Cu and Zn. TEM observations have revealed that this HEA thin film is being crystalline in nature with FCC and BCC phases. It has been reported that the single phase crystalline nature of the thin film with its symmetry can potentially reduce the *M*_*s*_ value by neutralizing some of the atomic magnetic moments^8^. Recent reports on HEAs show that soft magnetic nature is high in nanocrystalline materials as compared to the microcrystalline materials^8^. Though, the existing HEAs majorly show higher saturation magnetization, yet they often do not show lower coercivity simultaneously^8^. In spite of the presence of diamagnetic elements Cu and Zn in our system, the higher *M*_*s*_ and relatively lower *H*_*c*_ revealed at the same time in the present HEA is a positive outcome over existing HEAs which can be used as soft magnetic materials. Additional tuning of the electrodeposition parameters and relative composition of the constituent elements can further reduce the coercivity of the thin film. Moreover, the addition of oxidation/corrosion resistant elements like Ni, Cu and Zn in HEA thin films is expected to improve the oxidation resistance unlike the conventional magnetic materials made only from Co, Fe, Ni. It is also observed that HEAs show superior oxidation/corrosion resistant over conventional alloys^21,22^.

Thus the HEA thin films prepared by electrodeposition with a precise control over compositional space, microstructure and phase can result into various combination of properties. This can open innovative strategies in developing multiphase new class of HEAs as novel functional materials for several magnetic applications.

## Conclusion

Co–Cu–Fe–Ni–Zn nanocrystalline dual phase HEA thin films have been fabricated by pulse electrodeposition in a single step. Crystallinity, required compositional range of elements and phases in HEA thin films have been derived by the application of pulse in the electrodeposition. Where, pulse enabled the deposits with homogeneous distribution and composition throughout the film with dual phase of FCC and BCC having a nanocrystalline grain size ranging between ~ 5 and 20 nm. Impressive soft magnetic behaviour qualifies these HEA thin films as a novel functional material for several magnetic applications, where these HEAs are expected to have better corrosion/oxidation resistance over the conventional magnetic materials due to the presence of oxidation resistant elements in the existing alloy.

## Methods

Electrolyte composition for the preparation of these HEA thin films is given in (Supplementary Table S1). Area of deposition is 1 cm^2^ which is further scaled up to approximately 17 cm^2^ and their reproducibility also have been checked. Various combinations of pulse parameters were applied to achieve the range of compositions of alloys containing all five elements (atomic %). The optimized pulse parameters for preparing HEA thin films are given as forward ON time 50 ms; OFF time 10 ms; at 1.5 V in a potentiostatic mode using a Dynatronix pulse power supply. Since the deposition mechanism is not fully understood, probable correlations on the deposition process has been briefly explained based on our experimental observations. Microstructure and composition of the Co–Cu–Fe–Ni–Zn deposits were characterized by JEOL JSM 7800F field emission gun scanning electron microscope, equipped with an Energy dispersive X-ray spectroscopy (EDS) detector. The FCC-BCC dual phase HEA coating was confirmed by high-intensity 2D X-ray diffraction analysis using RAPID-II-D/MAX X-ray Diffraction System with high-intensity Cu-Cr dual wavelength rotating anode (RIGAKU Corp., Japan). A major FCC phase at a very small scales was confirmed by JEOL ARM-200F transmission electron microscope equipped with a Cs-corrector under an acceleration voltage of 200 kV. High angle annular dark field scanning TEM (HAADF-STEM) images were recorded and EDS spectra and elemental mapping were acquired as well. The magnetic properties of the thin films were characterized by vibrating sample magnetometry (VSM) and ferromagnetic resonance (FMR) techniques for determining the saturation magnetization, coercivity and remanent magnetization.

## Supplementary Information

Supplementary Figures and Tables

## Data Availability

The datasets generated during and/or analysed during the current study are available from the corresponding author on reasonable request.
