# Supplementary material for: An advancement in the synthesis of unique soft magnetic CoCuFeNiZn high entropy alloy thin films
Source: Sci Rep. 2021 Apr 23;11:8836. doi: 10.1038/s41598-021-87786-8 (PMC8065170; doi:10.1038/s41598-021-87786-8)
Supplement: Supplementary file 1 — Supplementary Figures and Tables [file 41598_2021_87786_MOESM1_ESM.pdf]

Supplementary Information

An advancement in the synthesis of unique soft magnetic CoCuFeNiZn high entropy alloy thin films

Chokkakula L. P. Pavithra[1,†], Reddy Kunda Siri Kiran Janardhana[1,†], Kolan Madhav Reddy[2], Chandrasekhar Murapaka[1], Joydip Joardar[3], Bulusu V. Sarada[3], Rameez R. Tamboli[1], Yixuan Hu[2], Yumeng Zhang[2], Xiaodong Wang[2], and Suhash Ranjan Dey[1,*]

[1]Department of Materials Science and Metallurgical Engineering, Indian Institute of Technology - Hyderabad, Sangareddy 502285, Telangana, INDIA.
[2]State Key Laboratory of Metal Matrix Composites, School of Materials Science and Engineering, Shanghai Jiao Tong University, Minhang District, Shanghai 200240, PR CHINA.
[3]International Advanced Research Centre for Powder Metallurgy and New Materials (ARCI), Balapur P.O., Hyderabad 500005, Telangana, INDIA.

* suhash@msme.iith.ac.in
† Authors with equal contribution

Composition/Parameters	***Range***
Iron (II) sulphate heptahydrate (FeSO$_4$·7H2O)	0.1 - 0.3 M
Cobalt (II) sulphate heptahydrate (CoSO$_4$·7H$_2$O)	0.05 - 0.4 M
Nickel (II) Sulphate hexahydrate (NiSO$_4$·6H$_2$O)	0.05-0.6 M
Nickel (II) chloride hexahydrate (NiCl$_2$·6H$_2$O)	<0.2 M
Copper (II) sulphate pentahydrate (CuSO$_4$·5H$_2$O)	0.0001 - 0.2 M
Zinc sulphate heptahydrate (ZnSO$_4$·7H$_2$O)	0.0005 - 0.2 M
Sodium citrate tribasic dehydrate (HOC(COONa)(CH2COONa)2·2H$_2$O)	0.001 - 0.1 M
Boric Acid (H$_3$BO$_3$)	0.1 - 0.7 M
Sulphuric Acid (H$_2$SO$_4$)/Sodium Hydroxide(NaOH)	to maintain pH
pH	2 - 3.5
Temperature	25 - 55ºC

Deposition duration	20 min
Thickness of the deposit	8-10 microns
Cathode	Titanium

Supplementary Table S1: Experimental Details*

*Order of mixing and duration between mixing of each salt in preparation of electrolyte plays a crucial role. This can change the pH and electrolyte stability at room temperature. Therefore, all the salts were dissolved separately and added one after the other and minimum duration for mixing is maintained as 3 hours between mixing of each single element electrolyte with other. Finally, boric acid is added.

Element	Total (at. %)	Error
**Fe**	24.7	3.7
**Co**	27.3	2.1
**Ni**	20.2	1.5
**Cu**	19.0	3.8
**Zn**	8.8	1.6

Supplementary Table S2: Average Elemental composition (atomic %) collected at various locations of the thin film

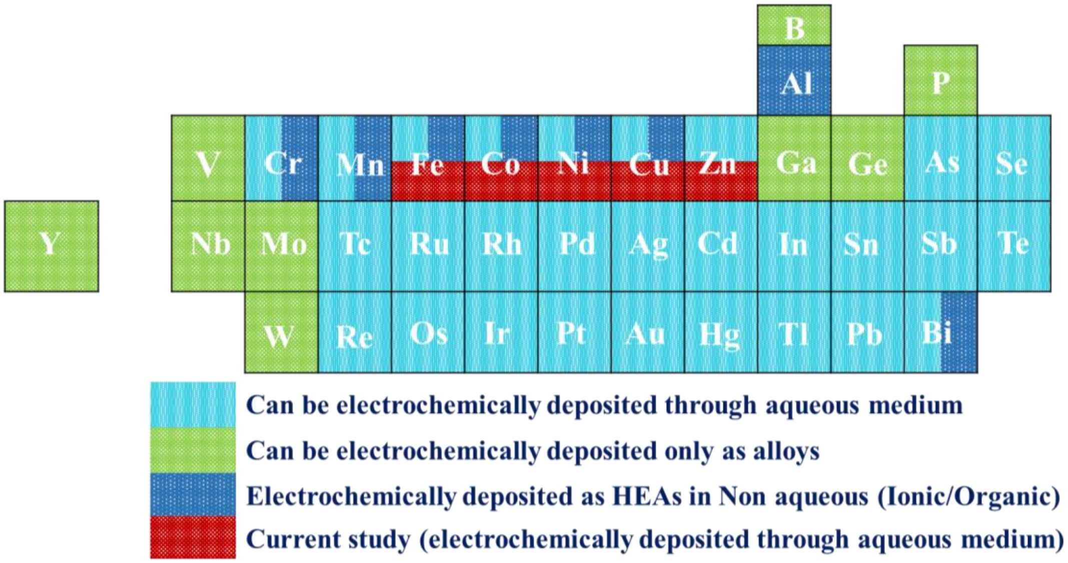

Supplementary Figure S1: Representation of elements in the periodic table explored for fabrication of high entropy alloy thin films by electrochemical approach

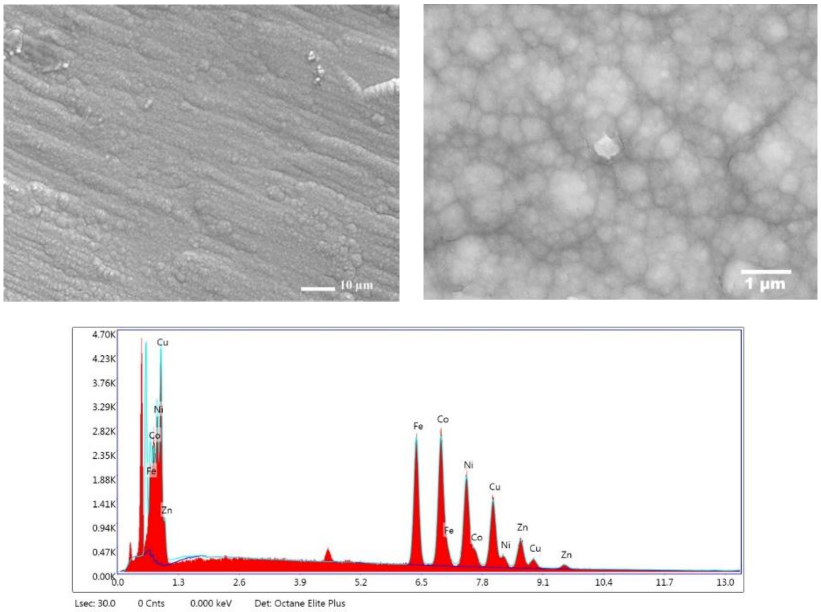

Supplementary Figure S2: FESEM micrograph of HEA thin film (at low and High magnification) and EDS

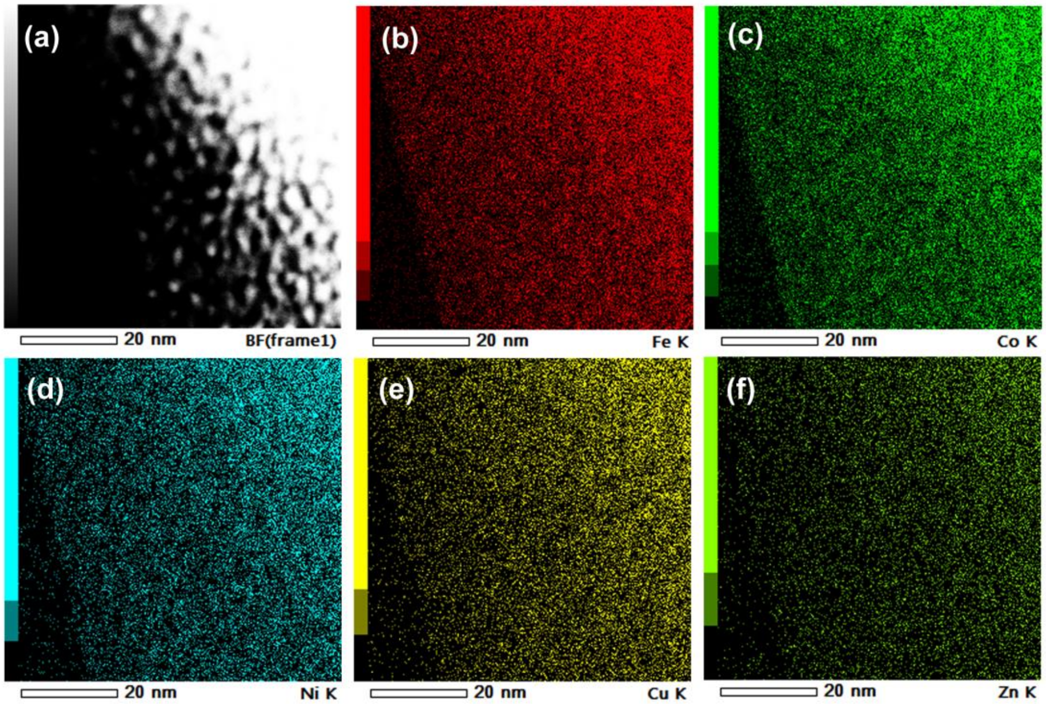

Supplementary Figure. S3: High resolution STEM-EDS and the corresponding elemental maps of Fe, Co, Ni, Cu and Zn.

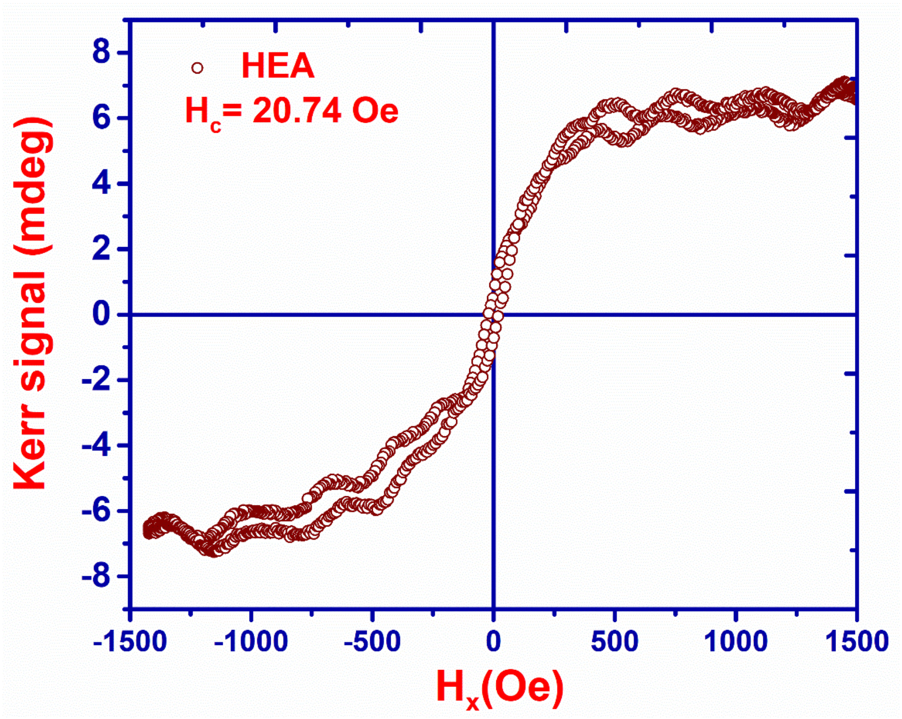

Supplementary Figure S4: Soft magnetic nature of the Co-Cu-Fe-Ni-Zn HEA thin film by MOKE (Magneto-optic Kerr effect measurement)

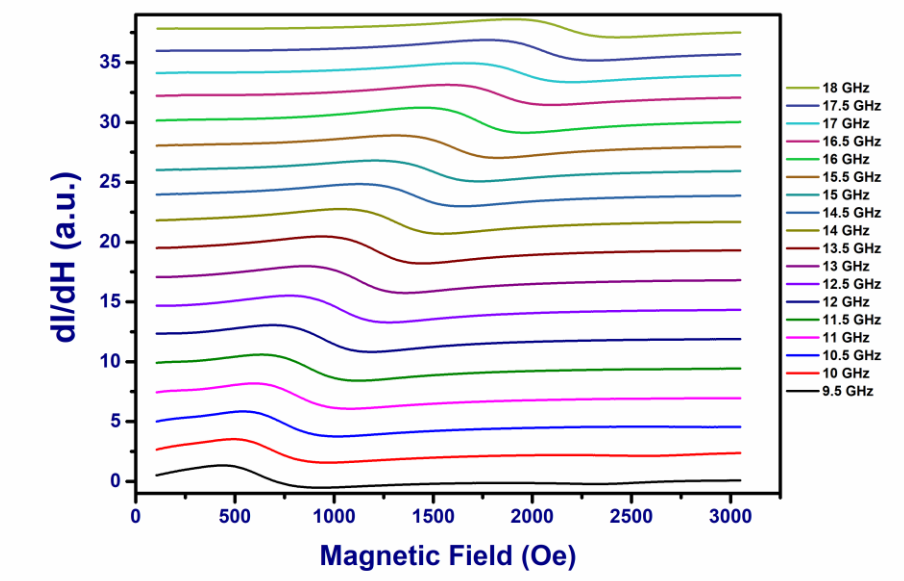

Supplementary Figure S5: FMR plots of Co-Cu-Fe-Ni-Zn HEA thin film
